# Epistemological Beliefs and Writing Self-Efficacy as Predictors of Second Language Writing Anxiety: A Structural Equation Modeling Approach

**DOI:** 10.3389/fpsyg.2022.850243

**Published:** 2022-04-25

**Authors:** Mohamad Heidarzadi, Hamed Barjesteh, Atefeh Nasrollahi Mouziraji

**Affiliations:** Department of English Language and Literature, Ayatollah Amoli Branch, Islamic Azad University, Amol, Iran

**Keywords:** epistemological beliefs, learners’ beliefs, structural equation modeling, writing anxiety, writing self-efficacy

## Abstract

This study was carried out to investigate the roles of epistemic beliefs (EBs) and writing self-efficacy (WSE) in predicting second language writing anxiety (L2WA) among learners of English as a foreign language (EFL). To this end, three validated scales were distributed among 240 EFL students. They were asked to complete the questionnaires during their regular courses. A structural equation modeling (SEM) approach was utilized to analyze the hypothesized SEM model and the causal paths among the constructs. The direct and indirect path analyses of the hypothesized model indicated that EBs and WSE accounted for 43% of the variance in L2WA. Although both constructs (i.e., EBs and WSE) had a significant effect on L2WA, EBs turned out to be a robust predictor of increasing L2WA. Notably, it was revealed that learners’ EBs directly and significantly influenced their L2WA. Besides, the results indicated that WSE had a unique effect in reducing L2WA. More precisely, students who had a higher level of EBs seemed to have a greater L2WA, and those who had a higher level of WSE experienced less L2WA. The findings of this explanatory study suggest that L2 teachers and material developers should pay serious attention to the Students’ cognitive and affective variables as they were known to be significant factors in influencing L2WA.

## Introduction

Second language (L2) writing is considered to be a cumbersome task. Numerous investigations postulate that writing skill is influenced by different cognitive, metacognitive, social, and psychological factors (Han and Hiver, 2018; Mitchell et al., 2019; Mitchell, 2020; Sun and Wang, 2020; Kärchner et al., 2021; Zhu et al., 2021). These factors make writing a demanding task and a dynamic activity. Therefore, L2 writers should consider how to utilize numerous strategies to promote the social cognitive process, communicate the ideas successfully, and manipulate their writing-specific psychological constructs (Lavelle, 2006; Bandura, 2011; Genc and Yayli, 2019; Barjesteh and Niknezhad, 2020; Mitchell, 2020). Various writing-specific psychological constructs [e.g., epistemic beliefs (EBs), second language writing anxiety (L2WA), writing self-efficacy (WSE), self-beliefs, self-concept, and context of writing] may influence learners’ writing performance by affecting their degree of engagement in generating effective writing (Bandura, 1977; Latif, 2007; Han and Hiver, 2018; Cheng, 2020). For effective writing, learners’ beliefs, the way they employ their thought about knowledge, how they view the quality of knowledge and learning (i.e., EBs), their tendency for initiating tasks, investing adequate effort to conduct activities, and endurance and perseverance in facing difficulties (i.e., self-efficacy) seem to be essential for planning a constructive language program (Bandura, 1977; Hofer and Pintrich, 1997). Extensive research (Bandura, 2011; Golombek et al., 2019; Sabti et al., 2019; Sun and Wang, 2020) has acknowledged that writing-specific psychological factors can facilitate or debilitate the writing process. Cheng (2004) explored L2WA as a writing-specific psychological construct that can debilitate writing achievement. Cheng considered L2WA as a facet of foreign language anxiety that provides a significant predictive effect on L2 writing. He explored different typologies of L2WA as a debilitative factor in the writing process. These typologies formulated the theoretical underpinning of this study. Hassan (2001) designated L2WA as “a general avoidance of writing and of situations perceived by the individuals to potentially require some amount of writing accompanied by the potential for evaluation of that writing” (p. 4). Such avoidance leads to “fear of the writing process that outweighs the projected gain from the ability to write” (Thompson, 1980, p. 121). The results of several theoretical and empirical studies substantiated the influence of self-beliefs on anxiety experience. Examining different dimensions of L2WA reveals that writing is a social and emotional activity, and WSE causes both direct and indirect contributions to the writing skill (Bandura, 2011). Some theoretical works (e.g., Bandura, 1977; Pajares, 2003; Rankin-Brown, 2006) authenticated such claim. Furthermore, some empirical studies (e.g., Cheng, 2004; Latif, 2007; Bruning et al., 2013; Mitchell, 2020) released evidence that the degree L2 writing self-beliefs may provoke anxiety experience among L2 writers. They considered WSE as a writing-specific psychological construct that may have predictive power in L2 writing. Bandura (1977) also pinpointed that learners’ sense of self-efficacy and self-beliefs on different language skills have a conspicuous role in performing the task. Lavelle (2006) explored that the degree of learner WSE can influence writing performance. He classified three degrees of WSE as high, mid, and low. He posited that students with high self-efficacy seem to have a positive sense of self in writing. A robust *sense of self-beliefs* in writing is called WSE (Bruning et al., 2013). Recently, some researchers and practitioners (e.g., Chen and Zhang, 2019; Sabti et al., 2019; Mitchell, 2020; Sun and Wang, 2020; Gloparvar and Khafi, 2021) endorsed that WSE positively contributes to L2 writing performance.

From a theoretical and empirical perspective, L2 professional literature also released evidence for the efficacy of EBs as a facet of a writing-specific psychological construct. The evidence of some empirical studies indicates that EBs have predictive power in some cognitive, social, and affective factors. Rezaei (2010) found the interplay between EBs and motivational beliefs in students with cognitive engagement. Winberg et al. (2019) examined the relationship between relationships between EBs and achievement goals. Shirzad et al. (2020) explored the relationship among EBs, language learning strategies, and L2 motivational self-system. In addition, the findings of Hofer and Pintrich (1997) supported the predictive effect of EBs in different aspects of education. Hofer (2016) outlined EBs as a psychological construct. Theoretically, the findings disclosed that learners with a high level of EBs seem to perform differently in language skills. Despite the extensive studies (e.g., Yang et al., 2019; Cheng, 2020; Shirzad et al., 2020; Kärchner et al., 2021; Zhu et al., 2021) on the predictive role of learners’ beliefs, there is a dearth of research on the interconnection among English as a foreign language (EFL) Students’ EBs, WSE, and WA in the L2 writing course. It has been hypothesized that EBs and WSE contribute to an increase in Students’ L2WA which in turn foster EFL learners’ writing achievement. In addition, it has been hypothesized that Students’ EBs with the mediating role of WSE may indirectly influence Students’ L2WA. Despite the sufficient evidence to support the positive effect of learners’ beliefs, this study argues that such constructs (i.e., EBs, WSE, and L2WA) have a complex and unpredictable relationship. Therefore, this study sought to test a model based on learners’ EBs and their WSE as the predictors of L2WA. Notably, this study was guided by the following objectives:

(i) To identify the relationship among Students’ EBs, WSE, and L2WA.
(ii) To determine whether epistemological beliefs positively predict Students’ L2WA.
(iii) To probe if WSE has a significant direct and indirect effect on L2WA.

## Literature Review

### Writing Anxiety

Researchers unanimously agree that the feeling of anxiety can negatively affect the performance of different language skills. This feeling in the context of education is called *foreign language anxiety.* Horwitz et al. (1986) conceptualized foreign language anxiety as the self-perception, emotion, and behavior concerned L2 learning arising from the particularity of the language learning process. MacIntyre and Gardner (1991) used the term *anxieties* instead of anxiety due to the specific nature of the task anxieties. Accordingly, foreign language anxiety is a type of task-specific anxiety. Cheng et al. (1999) acknowledged L2WA as “*language-skill specific anxiety*” (p. 417). Spielberger (1983) defined *anxiety* as “the subjective feeling of tension, apprehension, nervousness, and worry associated with an arousal of the autonomic nervous system” (p. 3). Researchers proposed reading, writing, listening, and speaking anxieties (Dörnyei, 2005). They considered language skill anxieties as a facet of foreign language anxiety. Likewise, some practitioners have suggested L2WA as a non-cognitive variable related to foreign language anxiety (Thompson, 1980; Hassan, 2001; Rankin-Brown, 2006; Latif, 2007). Hassan (2001) conceptualized L2WA as “a general avoidance of writing” (p. 4). Such avoidance leads to “fear of the writing process” (Thompson, 1980, p. 121). Thus, it would influence writing achievement. Cheng (2004) and Rankin-Brown (2006) have proposed different dimensions for L2WA. Cheng (2004) proposed three facets for L2WA, namely, *somatic anxiety, cognitive anxiety*, and *avoidance behavior.* Likewise, Rankin-Brown (2006) proposed four-dimensional aspects, namely, *fear of teacher and peer evaluation, fear of losing identity*, and *frustrations* due to self-evaluation. Other practitioners cited different sources (e.g., L2 WSE/self-confidence, L2 self-concept, perceived L2 writing performance, and L2 linguistic knowledge) that may provoke L2WA (Spielberger, 1983; MacIntyre and Gardner, 1991; Cheng et al., 1999; Latif, 2007). The theoretical foundation of this study is based on Cheng (2004) conceptualization of L2WA. Cheng hypothesized that L2WA emanates from (1) *somatic anxiety*, (i.e., learners’ attitudes toward unpleasant anxiety), (2) *cognitive anxiety* (i.e., the cognitive aspect of the anxiety), and (3) *avoidance behavior* (i.e., withdrawal/avoidance to act a particular task). The following Table 1 provides more detail of Cheng’s model for L2WA.

**TABLE 1:** Cheng’s (2004) three facets for second language writing anxiety (L2WA).

Construct	Definition	Task	Example(s)
Cognitive anxiety	Learners’ mental aspect when they experience anxiety	Negative expectations, preoccupation with performance, and concern about others’ perceptions.	Learners initiate negative self-related cognition
Somatic anxiety	Learners’ perception of the physiological effects of anxiety	Increase one’s autonomic arousal and unpleasant feeling states	Learners tend to sweat, shake, and increase their heart rate, suffer a headache, and rapid breathing
Avoidance behavior	The behavioral aspect when the learners are anxious	Learners with avoidance behavior	Learners will find ways and situations so that they do not have to write in English or to write compositions and to write compositions, not in the classroom

### Writing Self-Efficacy

The theoretical foundation of *self-efficacy theory* stemmed from Bandura’s unifying theory of behavioral change. Drawing on *social cognitive theory*, Bandura (1977) coined the term *self-efficacy* to show the expectation learners maintain about their ability to accomplish a task at specified levels. Bandura defined *self-efficacy* as a general psychological construct that delineates “people’s beliefs about their capabilities to produce designated levels of performance that exercise influence over events that affect their lives” (p. 71). Schunk (2012) clearly stated the concept of self-efficacy as one’s perception of his/her ability to do tasks at a specific level. When a task comes at the level of writing, a robust form of self-referent is called WSE (Bruning et al., 2013). Some studies (Pajares, 2007; Genc and Yayli, 2019; Sabti et al., 2019) suggested that dimensions of self-efficacy can influence learners’ motivation, goals, perseverance, and academic achievement. They confirmed that more specific formulated beliefs (e.g., beliefs about writing skills) have more predicative power than general formulated beliefs. It has also been illustrated that WSE is correlated with perceived academic self-efficacy, writing quality (Tsao et al., 2017), setting a goal for writing (Pajares, 2007), writing confidence (Mitchell et al., 2019), and writing motivation (Bruning et al., 2013). Moreover, other studies provided empirical evidence that the level of WSE can positively predict L1 writing attainment (Pajares, 2003; Cheng, 2004; Lavelle, 2006; Latif, 2007; Bandura, 2011; Bruning et al., 2013). Recently, some practitioners (e.g., Sun and Wang, 2020; Gloparvar and Khafi, 2021) have argued that studies on the cognitive and affective variables on the contribution of levels of self-efficacy beliefs to L2WSE have resulted in the void in the L2 professional literature. Bandura believed that the sources can be employed to display interventions directed to promote self-efficacy perceptions. Bandura (1977) distinguished four sources of self-efficacy, namely, (1) *performance accomplishments* (i.e., accomplishing a successful task in the past); (2) *vicarious experience* (i.e., the realization of social patterns that accomplish in performing a task); (3) *verbal persuasion* (i.e., the statements of others with accomplishing a task); and (4) *interpretation of emotional states* (i.e., aiding students to initiate proper clarifications of emotions they are experiencing). The strength of Bandura’s cognitive perspective is his distinction of how emotions are provoked (i.e., emotional arousal) in self-efficacy experiences, and how significant others (peers/teachers) can influence self-perceptions through modeling (i.e., vicarious experiences) and feedback provision (i.e., verbal persuasion). Later, Bruning et al. (2013) proposed some sources of self-efficacy to formulate the theoretical foundation of this study. They classified the sources as (a) *writing ideation* (i.e., learners’ beliefs about their ability for ideation in writing); (b) *writing conventions* (i.e., sense of self-beliefs about individual ability in performing linguistic rules to generate ideas); and (c) *writing self-regulation* (i.e., self-judgment about the individual capability to effectively manage writing process by different writing strategies such as setting writing objective, planning, maintaining writing motivation, and avoiding distractions). Bruning et al. (2013) proposed that writing ideation, writing conventions, and writing self-regulation have a predictive role in L2 writing performance.

### Epistemological Beliefs

Considerable studies (Schommer, 1990; Hofer, 2016; Al-Hoorie, 2018; Cheng, 2020; Dörnyei, 2020; Shirzad et al., 2020; Kärchner et al., 2021) in the area of learners’ beliefs attested that learners’ EBs can influence thinking processes, performance and competence, school love, monitoring, and learning strategies. By encroaching the concept of EBs in education, different models and conceptual frameworks have been developed, revised, and adapted by practitioners; however, the basic concept and core elements of EBs are almost the same (Hofer and Pintrich, 1997). Hofer (2016) defined EBs as learners’ ideologies about how knowing happens, what the nature of knowledge is, and how it is constructed and evaluated. Various professional researchers (e.g., Schommer, 1990; Hofer and Pintrich, 1997; White and Bruning, 2005; Tsao et al., 2017; Yang et al., 2019) unanimously agreed that EBs promote longitudinally from simple to complex thinking processes, while it may have direct and indirect effects on Students’ academic achievement. Accordingly, different dimensions of EBs have been proposed in the professional literature. Table 2 provides a historical overview of the way different authorities conceptualized EBs along with the various dimensions and main issues.

**TABLE 2:** Dimensions and general issues of epistemic beliefs (EBs).

Year	Authorities	Dimension	Main issue
1970	Perry	Dualism, multiplism, relativism, and commitment	Developmental sequence
1986	Belenky, Clinchy, Goldberger, and Tarule	Silence, received knowledge, subjective knowledge, procedural knowledge, and constructed knowledge	Exploring gender-related pattern in knowing
1990	Schommer	Omniscient authority, certain knowledge, simple knowledge, quick learning, and fixed ability	Personal epistemology Multidimensional, Independent
1997	Hofer and Pintrich	Certainty of knowledge (stability), simplicity (structure) of knowledge, source of knowing (authority), and justification for knowing (evaluation of knowledge claims)	Identifying dimensions of EBs

Initially, Perry (1970) shed light on EBs by proposing the concept for the later practitioners. He provided illuminated insight from a *developmental aspect* to delineate how a person improves through various stages to contrive EBs. Hofer (2000) suggested that EBs have two dimensions, namely, *nature of knowledge* [i.e., what one believes knowledge is (p. 380)] and the *nature/process of knowing* [i.e., how one comes to know (p. 380)]. Schommer (1990) criticized Perry’s model due to the one-dimensional facet. Schommer (1990) conceptualized EBs as a learner’s beliefs about knowledge and learning. She proposed a *multidimensional and independent* belief system containing five dimensions, namely, (1) *certainty* of knowledge (ranging from absolute to tentative); (2) *structure* of knowledge (from simple to complex); (3) *source* of knowledge (given by an authority or created by personal reasoning); (4) *control* of knowledge (fixed to changing and dynamic ability to learn something); and (5) *speed* of knowledge acquisition (quick to gradual knowledge acquisition). While the first three dimensions concern the nature of knowledge (certainty, structure, and source), the last two are related to acquisition/learning (control and speed). As a note of caution, Schommer’s personal epistemology yielded four dimensions, only in factor analysis results. This study was delimited into Students’ assumptions about the nature of knowledge (i.e., *simple/definitive knowledge*) and acquisition/learning (i.e., *fast/fixed learning agent*). Schommer’s first two dimensions were included in Hofer’s nature of knowledge. Schommer (1990) included beliefs about learning in EBs, while Hofer and Pintrich (1997) explicitly excluded such beliefs about learning. Historically, Students’ EBs have been examined by *self-reports, interviews*, and *open-ended questionnaires* (Perry, 1970; Schommer, 1990).

Schommer’s Epistemological Beliefs Questionnaire (EBQ) has been widely used as a well-known instrument for assessing learners’ EBs. Originally, EBQ comprised 63 items to be completed by the respondents. Some practitioners (Clarebout et al., 2001; Chan and Elliott, 2004; Rezaei, 2010) raised questions about the appropriateness of this scale to measure EBs. They remarked that the length of the items (*n* = 63) may demotivate the respondents to complete the questionnaire. Moreover, some items are difficult to understand. Therefore, a thorough revision of the questionnaire was reexamined to probe whether the use of EBQ is advisable in a setting different from the original one. Some empirical studies (Schommer-Aikins et al., 2000; Schraw et al., 2002; Chan and Elliott, 2004; Rezaei, 2010) have revisited Schommer’s questionnaire. They raised serious doubts on the practicality of the EBQ to measure EBs for different contexts. They examined the dimensionality of EBQ. In neither of these studies, the factor structure of Schommer (1990) could be retrieved. Notably, a different factor structure was found. They assumed that culture may be an influential factor in EB studies. Thus, some revised questionnaires have been developed and validated for different settings such as Japan, Turkey, Belgium, Netherlands, Chinese, Hong Kong, and Iran, to name but a few. They adapted Schommer’s questionnaire with the hope to be simplified, user-friendly, and compatible with the respondents’ culture and ethnicity. Accordingly, various dimensions and sub-factors have been proposed from the original version. The revised versions released fewer dimensions (e.g., two or three) and items (e.g., 16, 24, 30, and 42). Even, Schommer-Aikins et al. (2000) released different dimensions and items in their practical studies. The result of confirmatory factor analysis (CFA) revealed the loading of 30 items on different dimensions. Schommer-Aikins et al. (2000) proposed a three-dimensional questionnaire (i.e., speed, control, and certainty of knowledge acquisition) in the revised version. In addition, Clarebout et al. (2001) suggested another dimension of EBQ for Belgium and Dutch students. Similarly, Chan and Elliott (2004) adapted the questionnaire for Chinese and Hong Kong students. They offered a different dimension as compared with Schommer’s EBQ. Furthermore, Schraw et al. (2002) validated EBQ in a Turkish context. They analyzed EBQ without grouping 63 items into 12 subsets. To justify the dimensionality of EBQ, the professional literature (e.g., Clarebout et al., 2001; Schraw et al., 2002; Chan and Elliott, 2004; Shirzad et al., 2020) points to Schommer herself who regularly found deviant results while using the questionnaire. For the purpose of this study, an adapted version of the EBQ has been utilized. Rezaei (2010) examined the reliability and factor analysis of Schommer’s EBQ (1990) among 518 students studying at different majors. The result of reliability for the different subscales revealed that none of the reliability coefficients were acceptable. Thus, to examine the construct validity and to yield an appropriate factorial structure, factor analysis was conducted. Initially, the internal consistency was calculated for 63 items of EBQ. The results revealed that some items had a negative correlation (i.e., *n* = 11) or a correlation coefficient less than 0.1 (*n* = 17). Accordingly, a total of 27 items were extracted. Then, principal component analysis (PCA) was conducted on 36 items. The Scree plot of the primary analysis indicated that two factors should be generated for the analysis. Then, the Promax rotation method was performed in the PCA. The results revealed that 20 items either were below 0.35 or loaded equally under more than one factor during several rotations. Finally, 16 items were loaded under two dimensions. Based on the content of the items, they were classified into two dimensions, namely, (1) simple/absolute knowledge and (2) fast/fixed learning. Thus, the two dimensions were considered for examining the Students’ EBs for this study.

### Hypothesized Model

**FIGURE 1:**
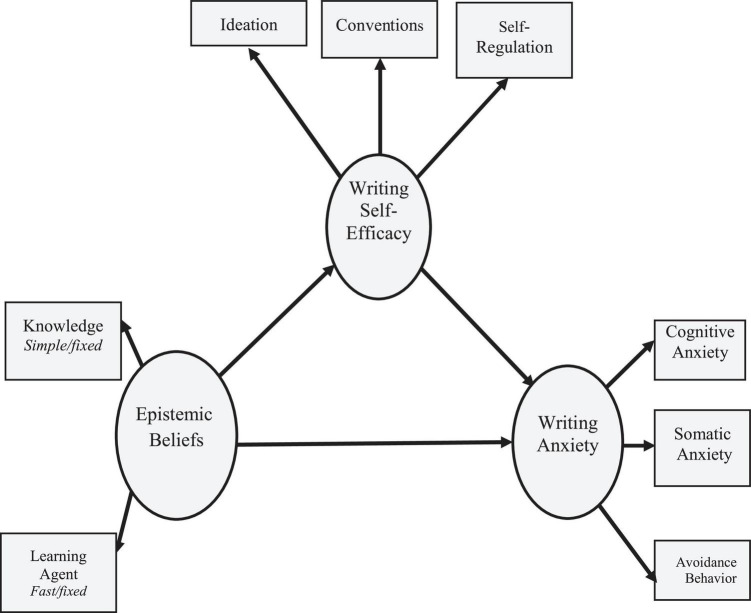
The hypothesized structural equation modeling (SEM) model and the causal paths among the variables.

The primary objective of this study was to examine a conceptual model of EBs and WSE as predictors of L2WA. In so doing, first, the unique contribution of either of the predictor variables is examined, and then, the concurrent contribution of the variables is explored. To undertake such an argument, this study substantiates a hypothetical model using a structural equation modeling (SEM) approach with mediating role of WSE. Figure 1 depicts the SEM framework and the interconnections among the latent and observed variables.

To test the hypothetical model, one null hypothesis and three directional hypotheses were formulated. Following this guideline, the subsequent research questions were investigated:

1. What is the nature of the relationship among Iranian EFL learners’ epistemological beliefs, WSE, and their writing anxiety?
2. Do epistemological beliefs have a significant direct effect on Students’ writing anxiety?
3. Does Students’ WSE have a significant direct effect on their writing anxiety?
4. Do epistemological beliefs with the mediating role of WSE have a significant indirect effect on writing anxiety?

## Materials and Methods

### Participants

The population of this study was comprised of 240 EFL students from different English language institutes in Tehran Province of the city of Karaj and Tehran in the autumn term 2019–2020 year. They were both men (*n* = 135) and women (*n* = 105) EFL learners whose ages varied from 20 to 25 years (*M* = 22.05; *SD* = 1.41) having already 3–5 years of experience in learning the English language at different English language institutes. The participants had the same native language (i.e., Persian), cultural, and social identity. A cluster random sampling method was adopted to select the target population. This was performed at the multistage cluster level. At the first level, a region was randomly selected from 5 distinct of Karaj and Tehran; the next 10 schools were randomly selected. Then, 25 clusters (i.e., online classrooms) were randomly selected. The participants were the same as far as their native language (i.e., Persian), cultural, social, and identity were concerned.

### Instruments

#### Assessing Students’ Epistemic Beliefs Toward Learning English

Students’ EBs were assessed by a revised version of Schommer (1990) EBQ. It comprised 16 items on a seven-point Likert-type response format. The EBQ has two dimensions opts for measuring Students’ (a) simple/definitive knowledge and (b) fast/fixed learning agent. The simple/definitive knowledge refers to the Students’ assumption about the nature of knowledge. The first dimension included 9 items (e.g., *scientists will ultimately get to the truth if they keep searching for it.; If scientists try hard enough, they can find the truth to almost anything; Anyone can figure out difficult concepts if one works hard enough; and wisdom is not knowing the answers, but knowing how to find the answers*). The second dimension concerns learners’ assumptions about acquisition/learning. The fast/fixed learning agent comprised 7 items (e.g., *Learning something really well takes a long time or much effort; How much you get from your learning depends mostly on your effort; If one tries hard enough, then one will understand the course material; Getting ahead takes a lot of work; Learning something really well takes a long time or much effort; and How much you get from your learning depends mostly on your effort*). The scales ranged from minimum (16) to maximum (112) scores. The EBQ scale enjoyed high reliability (α = 80.5) and validity indices at a pilot study among similar students (*n* = 90). The reliability coefficient for the subscale was as what follows: knowledge (simple/definitive) = 0.85 and learning agent (fact/fixed) = 0.76. As there is no generally accepted ordinal scale for measuring EBs, some studies (White and Bruning, 2005; Bendixen et al., 2010; Tsao et al., 2017; Shirzad et al., 2020) revealed that complex EBs are associated with higher moral reasoning and more complex thinking (e.g., knowledge is uncertain). Specifically, Bendixen et al. (2010) pinpointed that Students’ beliefs about simple knowledge, specific knowledge, and all-knowing authority are significantly connected with low moral justification and simpler views (e.g., knowledge is certain).

#### Second Language Writing Anxiety Inventory

To measure Students’ L2WA, Cheng (2004) inventory was employed. The inventory included 22 individual self-report items on a five-point Likert scale format. The inventory comprised three subscales including somatic anxiety (7 items), cognitive anxiety (8 items), and avoidance behavior (7 items). Somatic anxiety (items 2, 6, 8, 11, 13, 15, and 19) concerns the physiological effects of anxiety (e.g., *I feel my heart pounding when I write English; compositions under time constraint, My mind often goes blank when I start to work on an English composition; and I tremble or perspire when I write English compositions under time pressure*). Cognitive anxiety (items 1, 3, 7, 9, 14, 17, 20, and 21) deals with learners’ mental aspect (e.g., *While writing in English, I’m not nervous at all; While writing English compositions, I feel worried and uneasy if I know they will be evaluated, don’t worry that my English compositions are a lot worse than others*). Finally, avoidance behavior (items 4, 5, 10, 12, 16, 18, and 22) is a behavioral aspect when the learners are anxious (e.g., I often choose to write down my thoughts in English, I usually do my best to avoid writing English compositions, and I do my best to avoid situations in which I have to write in English). The higher score obtained from the inventory, the higher degree of psychological arousal, avoidance behavior, and fear concerned with L2 writing. The inventory enjoyed consistency reliability, respectable test–retest reliability (α = 0.91), and appropriate convergent and discriminant validity by means of correlation and exploratory factor analysis (EFA) (Cheng, 2004). For the purpose of this study, the estimated reliability was found to be (α = 0.83) among similar subjects (*n* = 68) in the EFL context of Iran. More precisely, the reliability coefficient for the subscale was as follows: somatic anxiety (α = 0.81), cognitive anxiety (α = 0.84), and avoidance behavior (α = 84.5).

#### Self-Efficacy Writing Scale

To determine EFL learners’ WSE, Bruning et al. (2013) scale was utilized. It is a multifactor perspective on WSE that aims to examine three tasks involved in the writing skill. The activities comprised 16 items in three subscales, namely, (1) the writing ideation (items 1–5), (2) the writing conventions (items 6–10), and (3) the writing self-regulation (items 11–16). The writing ideation aims to determine how learners generate ideas (e.g., *I can think of a lot of original ideas; I can think of many ideas for my writing; I can spell my words correctly*). The writing conventions determine how students express those ideas (e.g., *I can write complete sentences; I can write grammatically correct sentences; and I can focus on my writing for at least 1 h*). Finally, writing self-regulation manages writing decisions and behaviors (e.g., *I can avoid distractions while I write; I can start writing assignments quickly; and I can think of my writing goals before I write*). The scale takes response formats ranging from 0 (i.e., no confidence) to 100 (i.e., completely confident). The reliability and validity of the questionnaire were screened by Ramos-Villagrasa et al. (2018) among 512 male and female EFL learners from three different Spanish universities. Assessing internal consistency, the Cronbach’s α coefficient ranged from 0.89 to 0.90 for the different dimensions. The results of EFA endorsed that the self-efficacy writing scale kept its dimensionality in the Spanish version. More precisely, 89.66% of the variance was explained by the dimensions of the self-efficacy writing scale. The scale was translated into Persian and then back-translated into English. Then, three experts examined the translation with the original version. To assure the reliability of the scale within the EFL context of Iran, a pilot study was conducted among 98 EFL learners. The results enjoyed adequate reliability (α = 0.87). Specifically, each subscale enjoyed as appropriated reliability index as follows: (a) the writing ideation (α = 0.85), (b) the writing conventions (α = 0.89), and (c) the writing self-regulation (α = 0.87). The final items used for the analysis in each scale are presented in Supplementary Appendix.

### Procedure

To undertake the study, the SEM approach was adopted at two different subsequent phases, namely, the *preliminary phase* and the *main phase*. At the first stage, an EFA was employed to test direct and indirect relations among the dimensions of the target variables. Notably, it aimed to test the theoretically driven hypothesis about the linear interrelationships among the latent and observed variables. Then, three validated scales including Schommer (1990) EBQ, Cheng (2004) L2WA inventory, and Bruning et al. (2013) self-efficacy writing scales were distributed among the target participants. All the scales were digitally distributed *via* the Porcelain database during the pandemic COVID-19. The process of distribution was performed by the researcher who asked the respondents to answer as meticulously as possible. The students were asked to fill out the questionnaires at the same time during their regular class time. To have a valid response, they were assured the confidentiality of data. To minimize the bias effect, different EFL classes from different districts in two cities were randomly selected to distribute the questionnaires. To provide a valid response, the questionnaires were distributed to 240 students because some may be excluded from the sample due to the sample attrition. After collecting the data, all the responses were screened for fact-checking to promote the veracity and correctness of reporting. Consequently, a total of 24 questionnaires (10%) were not qualified for the analysis because they were incomplete or returned late. Notably, 216 questionnaires (90%) met a valid response rate of 95%. After collecting the valid responses, they were analyzed using SPSS and AMOS software using an SEM approach.

### Data Analysis

To undertake the study, a non-experimental correlational design was adopted. The analysis was carried out at different but interactive phases. First, descriptive statistics was run to measure the central tendency and to screen the statistical assumptions. Following the guidelines proposed by Hair et al. (2020), the subscale scores were examined for the outlier issues with univariate normality. Notably, a Mahalanobis test was run to eliminate the outlier data in the development of the linear regression model. To investigate the practicality, the hypothesized model Pearson correlation matrix, the CFA, and composite reliability (CR) were employed. Later, the SEM approach was run to test the predictive power of the latent and observed variables. Then, various goodness-of-fit indices (GFI) were checked. The GFI of the research variables was applied at three corrective steps comprising χ^2^/df (chi-square to degrees of freedom ratio), root mean square error of approximation (RMSEA), Tucker-Lewis index (TLI), and comparative fit index (CFI). Hair et al. (2020) suggested that the acceptable ranges for values of such indices are χ^2^/*df* < 3, TLI > 0.95, GFI > 0.95, RMSEA < 0.06, and CFI > 0.95.

## Results

### Screening the Assumptions of Normality

To answer the research questions, some basic steps were conducted to check the assumptions for the normality of the constructs. The values for skewness and kurtosis for all subscales fall within the acceptable range. The normal range of values obtained in this test is between −2 and 2. Table 3 reveals skewness, kurtosis, and normality analysis for the subscales of the constructs.

**TABLE 3:** Skewness, kurtosis, and normality test for different variables.

	Construct skewness		Kurtosis		Kolmogorov-Smirnov^a^	
	SE	Statistic	SE	Statistic	Statistics	Sig
K	0.153	0.138	−0.855	0.311	1.361	0.049
LA	0.389	0.138	−0.562	0.311	1.452	0.030
EBs	0.785	0.138	−0.001	0.311	1.449	0.030
WI	–0.112	0.138	−0.396	0.311	1.396	0.041
WC	–0.692	0.138	−0.125	0.311	1.372	0.046
WSR	0.785	0.138	−0.753	0.311	1.055	0.215*
WSE	–0.055	0.138	−0.693	0.311	1.434	0.033
SA	0.350	0.138	−0.96	0.311	1.785	0.004
CA	0.669	0.138	−0.107	0.311	1.621	0.018
AB	–0.741	0.138	−280	0.311	1.458	0.032
L2WA	–0.520	0.138	−0.452	0.311	1.521	0.034

*k, knowledge; LA, learning agent; WI, writing ideation; WC, writing convention; WSR, writing self-regulation.*

^a^Lilliefors significance correction. *This is a lower bound of the true significance.

Referring to Table 3, the data seem to satisfy the assumption of normality. Notably, the values for skewness and kurtosis for all subscales were within acceptable ranges following the guidelines proposed by Aryadoust and Raquel (2020). More precisely, the measure of skewness (range = −0.055 to 0.785) and kurtosis (range = −0.001 to −0.855) are at appropriate bound for the different subscales. In addition, the distribution of data was not normal as the result of Kolmogorov-Smirnov test (*p* > 0.05). To identify multivariate outlier data, the Mahalanobis test was performed. Table 4 indicates the outlier data.

**TABLE 4:** Outlier detection with Mahalanobis distance.

	Minimum	Maximum	Mean	SD	N
Mahalanobis’ distance	0.132	52.85	11.175	2.693	240
Leverage values	0.000	0.008	0.004	0.003	240

The subscale scores were examined for the outliers and the issues with univariate normality. There were 24 extreme ratings (*k >* 24.99) identified from a possible rating (i.e., *Min* = 132; *Max* = 52.85). Accordingly, the univariate outliers were extracted from the data set. In brief, the Mahalanobis distance (MD) analysis at a critical α value of 0.005 indicates that 24 multivariate outliers do not match the general character of the dataset, and a total of (*n* = 216) students met the normal range. The analyzing different assumptions for the normality of the data (e.g., Kolgomorov–Smirinov, Mahalanobis test, Q-Q plot, and P-P plot) revealed that the data are normal and leaving a safe result for the inferential statistics.

### Testing the Proposed Model: Preliminary Analysis for Construct Validity

**FIGURE 2:**
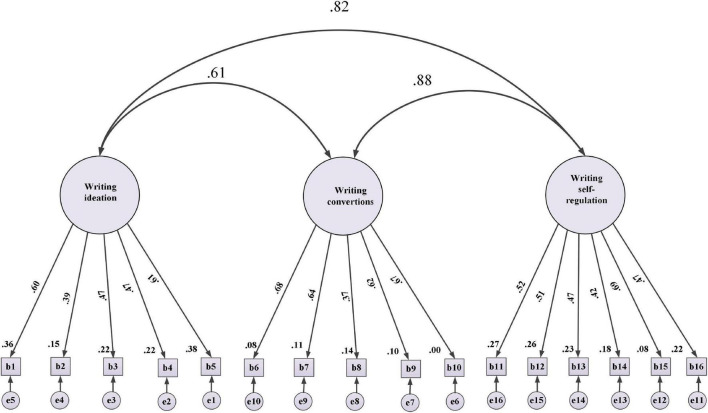
Standardized (β) coefficients for confirmatory factor analysis (CFA) and error variance for writing self-efficacy (WSE).

**FIGURE 3:**
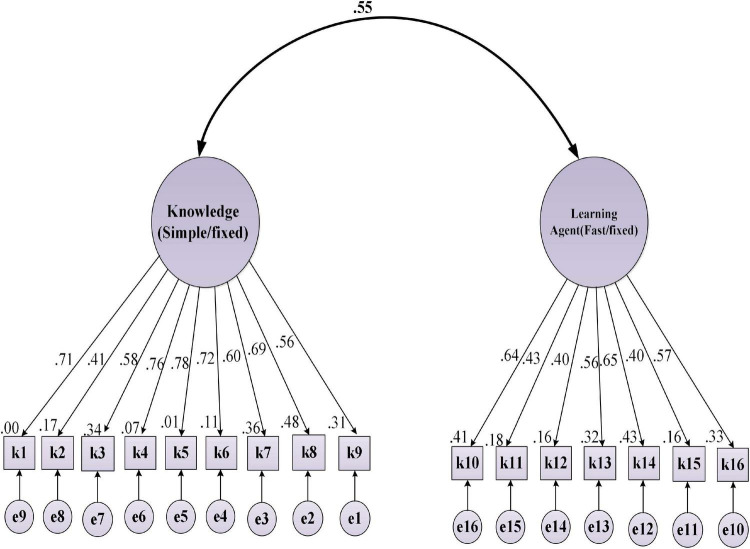
Standardized (β) coefficients for CFA and error variance for epistemic beliefs (EBs).

**FIGURE 4:**
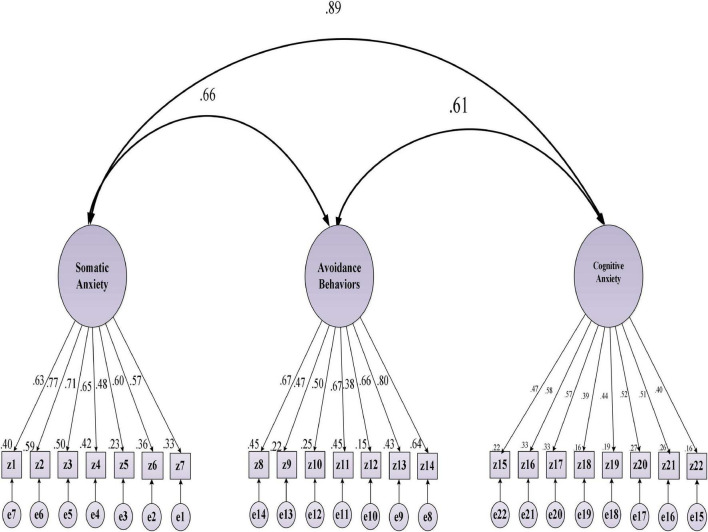
Standardized (β) coefficients for CFA and error variance for second language writing anxiety (L2WA).

To assure the construct validity of the target scales, CFA was performed. Kline (2011) suggested this preliminary analysis before running the SEM to check the measurement model. Figures 2–4 represent the standardized beta (β) coefficients for CFA and error variance of the constructs (i.e., WSE, EBs, and L2WA). This value indicates the slope of the line between the dependent variable (L2WA) and the predictors (i.e., WSE and EBs). Besides Tables 5–7 indicate the goodness of fit indices of the each variable before revision.

**TABLE 5:** Goodness-of-fit indices of the writing self-efficacy (WSE) before revision.

Fit index		Preference value	Obtained value	Result
	X^2^/degree of freedom	Nil	184.392	−
AGF	CFI	≥0.90	0.994	Goodness of fitting
	AGFI	≥ 0.90	0.982	Goodness of fitting
	NFI	≥0.90	0.990	Lack of fit
	CFI	≥ 0.90	0.993	Goodness of fitting
	RFI	≥0.90	0.997	Lack of fit
	PNFI	0.5 ≤	0.528	Goodness of fitting
	SRMR	0.08 ≥	0.042	Standardized root mean square residual
PFI	RMSEA	0.08 ≥	0.039	Root mean square error of approximation
	Degree of freedom/X^2^	3 ≥	2.561	Lack of fit
	Df	≤	72	−
	P	0.05 ≥	0.001*	Goodness of fitting

*p < 0.05.

**TABLE 6:** Goodness-of-fit indices of the EBs before revision.

Fit index		Preference value	Obtained value	Result
	X^2^/degree of freedom	Nil	112.098	−
AGF	CFI	≥0.90	0.994	Goodness of fitting
	AGFI	≥0.90	0.991	Goodness of fitting
	NFI	≥0.90	0.999	Lack of fit
	CFI	≥0.90	0.997	Goodness of fitting
	RFI	≥0.90	0.991	Lack of fit
	PNFI	0.5≤	0.536	Goodness of fitting
	SRMR	0.08≥	0.040	Standardized root mean square residual
PFI	RMSEA	0.08≥	0.034	Root mean square error of approximation
	Degree of freedom/X^2^	3≥	2.198	Lack of fit
	Df	≤	51	−
	P	0.05≥	0.001*	Goodness of fitting

*p < 0.05.

**TABLE 7:** Goodness-of-fit indices of the L2WA before revision.

Fit index		Preference value	Obtained value	Result
	X^2^/degree of freedom	Nil	222.357	−
AGF	CFI	≥0.90	0.990	Goodness of fitting
	AGFI	≥0.90	0.986	Goodness of fitting
	NFI	≥0.90	0.991	Lack of fit
	CFI	≥0.90	0.990	Goodness of fitting
	RFI	≥0.90	0.994	Lack of fit
	PNFI	0.5≤	0.511	Goodness of fitting
	SRMR	0.08≥	0.044	Standardized root mean square residual
PFI	RMSEA	0.08≥	0.040	Root mean square error of approximation
	Degree of freedom/X^2^	3≥	2.679	Lack of fit
	Df	≤	83	−
	P	0.05≥	0.001*	Goodness of fitting

*p < 0.05.

The standardized (β) coefficients for CFA and error variance of the constructs indicated that the fit model was above 0.30. Notably, all the sub-factors in WSE have the predictive power (>0.30; *df* = 89; RMSEA = 0.031; sig = 0.000; GFI = 0.971; CFI = 0.989; NF = 9.91). Measuring CFA for EBs confirmed that the factor loading of all subscales for EBs are above 0.30 indicate good fit (i.e., df = 62; RMSEA = 0.033; sig = 0.000; GFI = 0.989; CFI = 0.990; NFI = 0.996; *p* < 0.05). In addition, all observed variables for the L2WA are indicative of acceptable model fit (i.e., df = 128; RMSEA = 0.031; sig = 0.000; GFI = 0.991; CFI = 0.979; NFI = 0.988). Notably, the value confirms that the factor loading of all subscales for L2WA indicates the fitness level for the corresponding model. To measure the internal consistency of the items in the target variables, the CR was performed. CR (sometimes called *construct reliability*) is the ratio of the variance of the latent and a linear combination of the latent variable ranging from 0.7 to 0.95 (Hair et al., 2020). To check the degree of shared variance between the latent variables, Fornell and Larcker (1981) criterion is commonly considered. Accordingly, the convergent validity of the measurement model was examined by the average variance extracted (AVE) and CR. Fornell and Larcker suggested that the CR index value should exceed 0.7, and the minimum AVE index values should exceed 0.5. Table 8 reports the CR analysis between constructs and the corresponding sub-factors.

**TABLE 8:** Composite reliability analysis for constructs.

No.	Construct	Cronbach alpha	AVE (*p* > 0.5)	CR (*p* > 0.7)
1	K	0.711	0.521	0.718
2	LA	0.796	0.630	0.749
3	EBs	0.782	0.502	0.752
4	WI	0.813	0.587	0.736
5	WC	0.796	0.541	0.788
6	WSR	0.805	0.598	0.720
7	WSE	0.839	0.562	0.796
8	SA	0.769	0.510	0.824
9	CA	**0.682**	0.577	0.715
10	AB	0.726	0.539	0.763
11	L2WA	0.775	0.524	0.784

*Construct: 1. K, knowledge; 2. LA, language anxiety; 3. EBs, epistemic beliefs; 4. WI, writing ideation; 5. WC, writing convention; 6. WSR, writing self-regulation; 7. WSE, writing self-efficacy; 8. SA, somatic anxiety; 9. CA, cognitive anxiety; 10. AB, avoidance behaviors; 11. L2WA, second language writing anxiety.*

Table 8 reveals that the internal consistency for all the subscales was good to excellent (*Cronbach’s α range* = 0.711–0.839) except for the scale which shows relatively low test score reliability (α = 0.682). Accordingly, all scales reported internal consistency as far as AVE and CR were concerned.

### Assumption Testing for the Pearson Product-Moment Correlation

Pearson product-moment correlation was conducted among the main variables and the corresponding sub-factors of this study. Table 9 indicates the Pearson correlation matrix among EBs, L2WA, and WSE.

**TABLE 9:** Pearson correlation matrix among the constructs and the sub-factors.

C*	M	SD	1	2	3	4	5	6	7	8	9	10	11
1	24.39	4.11	1										
2	19.05	4.68	0.69**	1									
3	42.63	8.52	0.77**	0.81**	1								
4	12.39	2.87	−0.21**	−0.16**	−0.26**	1							
5	14.51	2.69	−0.19**	−0.20**	−0.24**	0.62**	1						
6	15.07	3.40	−0.20**	−0.19**	−0.28**	0.55**	0.68**	1					
7	48.20	7.56	−0.22**	−0.25**	−0.31**	0.71**	0.77**	0.82**	1				
8	17.29	3.58	0.23**	0.19**	0.26**	−0.19**	−0.18**	−0.21**	−0.22**	1			
9	20.47	5.41	0.29**	0.25**	0.22**	−0.17**	−0.19**	−0.17**	−0.26**	0.72**	1		
10	18.13	4.20	0.24**	0.21**	0.28**	−20**	−0.21**	−0.19**	−0.28**	0.65**	0.68**	1	
11	62.91	11.78	0.33**	0.28**	0.35**	−0.23**	−0.25**	−0.23**	−0.34**	0.81**	0.73**	0.68**	1

*Construct: 1. K, knowledge; 2. LA, language anxiety; 3. EBs, epistemic beliefs; 4. WI, writing ideation; 5. WC, writing convention; 6. WSR, writing self-regulation; 7. WSE, writing self-efficacy; 8. SA, somatic anxiety; 9. CA, cognitive anxiety; 10. AB, avoidance behaviors; 11. L2WA, second language writing anxiety. **p < 0.01.

As demonstrated in Table 9, there is a significant linear relationship among EBs, WSE, and L2WA. Notably, there is a significant linear relationship between the components of EBs (i.e., simple/definitive knowledge, fast/fixed learning agent, and the total score) and the total score of WSE with respect to the sub-factors of L2WA. In particular, there is a significant positive relationship between two constructs (i.e., EBs and L2WA) and the corresponding sub-factors. The finding shows that when EFL learners’ EBs promote, their L2WA will increase subsequently. Furthermore, there is a significant negative relationship between WSE and L2WA. More precisely, EFL learners’ WSE will help them reduce their L2WA.

### Analysis of Direct and Indirect Effects of Epistemic Beliefs and Writing Self-Efficacy on Second Language Writing Anxiety

To examine the overall model fit and to check the predictive power of the independent variables on the dependent variable, various GFIs have been examined. As the vital part of the SEM approach, Hair et al. (2020) suggested different indices, including χ^2^/df, GFI, RMSEA, TLI, and CFI. Thus, the preference value for each index was considered for the evaluation. Table 10 indicates the goodness-of-fit indices for the variables of this study.

**TABLE 10:** Goodness-of-fit indices of the research variables before revision.

Fit index		Preference value	Obtained value	Result
	X^2^/degree of freedom	Nil	557.407	−
AGF	CFI	≥0.90	0.898	Goodness of fitting
	AGFI	≥0.90	0.971	Goodness of fitting
	NFI	≥0.90	0.891	Lack of fit
	CFI	≥0.90	0.945	Goodness of fitting
	RFI	≥0.90	0.881	Lack of fit
	PNFI	0.5≤	0.509	Goodness of fitting
	SRMR	0.08≥	0.048	Standardized root mean square residual
PFI	RMSEA	0.08≥	0.051	Root mean square error of approximation
	Degree of freedom/X^2^	3≥	3.013	Lack of fit
	Df	≤	185	−
	P	0.05 ≥	0.001*	Goodness of fitting

*p < 0.05.

Table 11 reveals three fit indices [i.e., Adjusted Goodness of Fit Indices (AGFI), CFI, and Parsimonious Fit Index (PFI)] for the research variables. As indicated in Table 11, the obtained values for the hypothesized model are below 0.90 representing the lack of fit for the model. Thus, the hypothesized model needs modification. Table 11 indicates the GFI of the research variables after applying all modifications suggested by AMOS.

**TABLE 11:** Goodness-of-fit indices of the research variables after two stepwise corrections.

Fit index		Preference value	Obtained value	Result
	X^2^/degree of freedom	Nil	521.733	−
AGF	GFI	≥0.90	0.995	Goodness of fitting
	AGFI	≥0.90	0.989	Goodness of fitting
	NFI	≥0.90	0.956	Goodness of fitting
	CFI	≥0.90	0.988	Goodness of fitting
CFI	TLI	≥0.90	0.969	Goodness of fitting
	RFI	≥0.90	0.944	Goodness of fitting
	PNFI	0.5≤	0.531	Goodness of fitting
PFI	RMSEA	0.08≥	**0.039**	Goodness of fitting
	Degree of freedom/X^2^	3≥	2.851	Goodness of fitting
	Df	≤	183	−
	*P*	0.05≥	0.001*	Goodness of fitting

*p < 0.05.

After the modification of the basic model proposed using the AMOS software, a revised model was suggested. The model was finalized after two stepwise corrections in order to fall within the normed fit index. Table 11 indicates (RMSEA (0.039 < 0.08). Notably, the mean square error of the revised model falls within the preference value. Besides, the chi-square value (X^2^ = 2.851) is between 1 and 3 preference value. Likewise, the fit indices of GFI, CFI, AGFI, NFI, and TLI are also all above the critical point (≥0.90; GFI = 0.995; AGFI = 0.989; NFI = 0.956; CFI = 0.988; TLI = 0.969; and RFI = 0.944). The findings confirm the normal fit index. Thus, the revised measurement model was appraised applicable for further analysis. Table 12 reveals the regression analysis and coefficients for the exogenous and indigenous variables.

**TABLE 12:** Regression analysis and coefficients*^a^* for exogenous and indigenous variables.

Exogenous variable	Direction	Indigenous variable	Unstandardized coefficients	Standardized coefficients	t	Sig.
			B	β		
EBs	→	L2WA	0.457	0.325	4.215	0.001
WSE	→	L2WA	−0.425	−0.304	3.961	0.001

*^a^Dependent variable: L2WA.*

As displayed in Table 12, the predictor variables (i.e., EBs and WSE) have a low *p*-value (*p* < 0.05). In other words, the changes in the predictors/exogenous variables are associated with changes in the response value. More specifically, the coefficient for exogenous variables (i.e., EBs and WSE) is as follows: EBs (*t* = 4.215) and WSE (*t* = 3.961). They are statistically significant since the *p*-value is smaller than the significance level (*p* < 0.05). The finding reveals that all the exogenous variables are the robust predictor of L2WA. Accordingly, the following directional hypotheses are proposed:

**H_1_1:** EBs directly affect Students’ L2WA.
**H_1_2:** WSE has a significant direct effect on Students’ L2WA.
**H_1_3:** EBs with the mediating role of WSE have a significant indirect effect on the L2WA.

To determine the direct effect of EBs and WSE on L2WA, maximum likelihood estimation (MLE) was adopted. MLE is a method of evaluating the parameters of distribution by promoting a likelihood function (Richard, 2018). Table 13 indicates the result of MLE for WA.

**TABLE 13:** Direct maximum likelihood estimation for L2WA.

Variable	Unstandardized coefficients	Standardized coefficients			
	B	β	*R* ^2^	t	Sig.
EBs	0.457	0.325	0.148	4.215	0.000
WSE	−0.380	−0.246	0.096	3.215	0.000

As indicated in Table 13, the standardized coefficients for EBs and WSE are β_*EBs*_ = 0.325 and β_*WSE*_ = −0.246, respectively. Furthermore, *R*^2^ for the corresponding variables are *R*^2^_*EBs*_ = 0.148 and *R*^2^_*WSE*_ = 0.096). The results obtained from standardized coefficients (β) and *R*^2^ confirmed that the conceptual model proposed is statistically significant. Thus, the assumptions for the finalized research model between latent and observed variables have not been violated. To determine if the EBs indicate a reverse effect on L2WA with the mediating role of WSE, the bootstrapping regression model was employed. The bootstrap is a versatile method for estimating the sampling distribution of parameter estimates in order to test the indirect effects. Bootstrapping is a method that resamples a single dataset to generate various simulated samples (Aryadoust and Raquel, 2020). Table 14 indicates the bootstrap estimate of indirect effect with a mediating role of WSE on L2WA.

**TABLE 14:** Bootstrap estimate of indirect effect with mediating WSE.

Variable	β	Lower limit	Upper limit	Sig.
EBs with mediating WSE on L2WA	−0.541	−0.612	−0.395	0.001

**FIGURE 5:**
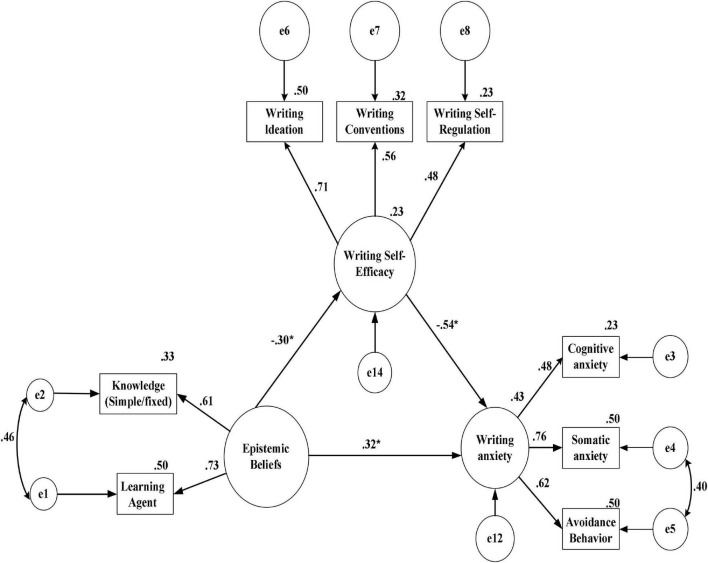
Finalized unstandardized test for direct and indirect paths in predicting L2WA with respect to WSE and L2WA. *p < 0.05.

Table 14 shows the standardized β coefficients (β = −0.541, the lower limit = −0.612; upper limit = −0.395). The results indicate that EBs with mediating WSE on L2WA are significant with respect to the bootstrap regression model. Figure 5 schematically represents the finalized model tested with the unstandardized and standardized statistics. Notably, this figure illustrates the direction in predicting L2WA with respect to WSE and L2WA.

As indicated in the final model, the subscale variables have factor loading above (>0.30). This proved that the sub-factors are compatible with the latent variables. Moreover, the covariances in the sub-factors of EBs and L2WA enjoy acceptable fit indices for the revising model. The EBs could also account for 0.23 of variance in WSE. Figure 5 indicates that EBs had a significant direct effect on L2WA. Besides, EBs had a significant indirect effect on L2WA through the effects of WSE. As a result, it can be argued that WSE serves as a partial mediator in the relationship between EBs and L2WA. The direct and indirect paths could account for 43% of the variances in L2WA.

## Discussion

The purpose of this study was to examine the significance of EFL learners’ EBs and WSE in predicting learners’ L2WA. More specifically, the interconnections among the components of the three constructs comprising eight sub-factors were investigated using an SEM modeling approach. The results of SEM analyses revealed that both constructs (i.e., EBs and WSE) had a different contribution to L2WA. The standardized paths after correction for direct and indirect analyses revealed a direct and indirect effect on EFL learners’ L2WA with respect to the exogenous factors (i.e., EBs and WSE). Notably, the findings indicated that there is a significant linear interplay among the constructs (i.e., EBs, WSE, and WA). In particular, there is a significant positive relationship between EBs and L2WA. The path analysis of the hypothesized model reveals that EBs have a direct significant effect on L2WA, and they are a robust predictor for such variables. In particular, learners’ EBs directly increase EFL learners’ L2WA. This shows that the level of EFL learners’ EBs can increase their L2WA. The findings echo different bodies of studies (e.g., Hofer, 2016; Al-Hoorie, 2018; Yang et al., 2019; Shirzad et al., 2020). Specifically, the findings confirmed that the level of learners’ EBs affects the type of strategies they utilize in language learning. In general, they have noted that learners’ EBs can promote their academic achievement both directly and indirectly. Notably, they found that when learners imagined themselves as competent people, they can use less learning strategy. Particularly, this finding echoes Schommer (1990) theoretical claim that various aspects of the beliefs about the structure and source of knowledge affect learners’ academic achievement and psychological factors.

The findings are consistent with the theoretical and empirical models (Hofer and Pintrich, 1997; Hofer, 2016; Winberg et al., 2019) proposed in the L2 professional literature. They released that some dimensions of EBs are the negative predictor of academic achievement. In particular, they confirmed that if learners consider the scientific issues as inherent and unambiguous facts, their academic achievement will decrease accordingly. To endorse this claim, Shirzad et al. (2020) concluded that the beliefs held by the learners affect the way they plan to study. Likewise, the standardized model and the directional and non-directional paths of the model proposed in this study revealed that there is a significant positive interplay between learners’ EBs and their L2WA. In brief, the more the level of learners’ EBs in language learning, the higher anxiety they may experience in L2 writing.

The second phase of this study was to probe the predictive role of WSE in L2WA. It was set to investigate how the level of learners’ WSE can promote or demote their writing performance. Notably, the hypothesized model proposed in this study aimed to uncover if EFL learners’ WSE has a significant direct effect on their L2WA. The results revealed that the correlation between learners’ WSE and L2WA was significant. The path analysis of the proposed model revealed that WSE had a direct significant effect on L2WA. Particularly, the findings indicated that there is a significant negative relationship between WSE and L2WA. To put it exactly, the findings confirmed that WSE had a significant direct effect in reducing EFL learners’ L2WA. As a significant interplay between Students’ self-efficacy beliefs and their writing performance was found in an accumulated body of studies (e.g., Bandura, 1994; Pajares, 2003; Cheng, 2004; Lavelle, 2006; Latif, 2007), the findings also endorsed such a claim that WSE influences writing performance. The results showed that the components of WSE had a significant predictive power on L2WA. In detail, the results of correlational analyses indicated that learners’ WSE was inversely correlated with L2WA. This is in line with various studies (e.g., Bandura, 1977; Lavelle, 2006; Latif, 2007; Pajares, 2007) reporting that more degree of self-efficacy may lead to less degree of affective factors. Currently, some practitioners (e.g., Mitchell et al., 2019; Barjesteh and Niknezhad, 2020; Sun and Wang, 2020; Gloparvar and Khafi, 2021; Kärchner et al., 2021) released evidence that students may perform better in writing when they feel more self-confidence in their competency to write. Similarly, this study endorsed such a claim by the analysis of the causal paths between WSE and L2WA. Specifically, the hypothesized SEM model pinpointed that when EFL learners’ WSE increases, students can perform better in their writing skills. This is due to the fact that the level of Students’ WSE can negatively affect their L2WA. To put it differently, when learners’ WSE increases, their L2WA will decrease in the end.

Finally, the last phase of this study was to test the directional hypothesis claiming that EBs with mediating role of WSE have a significant indirect effect on L2WA. The analysis of bootstrapping regression model pinpointed that EBs have a reverse effect on L2WA. The findings confirmed that WSE served as a mediator. The results revealed that the independent variables (i.e., EBs and WSE) could account for 43% of the variance in L2WA. This finding echoes Pajares and Valiente (1997) who found that WSE directly influenced learners’ anxiety. Pajares and Valiente found that WSE affects feelings about its perceived usefulness, and the assessment of essay writing. The results are in line with Bandura (2011) argument which postulated that learners’ efficacy beliefs affect the activities they perform, the thinking patterns they have, and the emotions they postulate. In line with Bandura’s argument, the findings indicated that L2 writers enjoy writing when they feel self-belief (i.e., self-confidence) in their ability to write. Likewise, White and Bruning et al. (2013) posited that learners’ WSE influences the quality of their writing. They advised that writing teachers should consider the LSE perceptions of the students so that “*integrated models of writing*” (p. 186) can be incorporated to meet EFL learners’ writing needs. Similarly, Lavelle (2006) concluded that learners with high self-efficacy levels suggest the hard writing task as a challenging task to accomplish by making contributive use of their cognitive strategies. More recently, the finding is in line with Tsao et al. (2017) who claimed that the level of learners’ anxiety correlates with writing in English. Similarly, Genc and Yayli (2019) concluded that SA was the most common type of anxiety, avoidance anxiety comes second, and cognitive anxiety is the last anxiety type among EFL learners. The findings of this study also support Sabti et al. (2019) who found a negative relationship between WSE and writing anxiety among students. The findings add some points in the L2 literature by endorsing authorities’ claim (e.g., Cheng et al., 1999; Han and Hiver, 2018; Mitchell et al., 2019; Sun and Wang, 2020) that L2 writers should consider different social, cognitive, and communicative demands in the writing process. The findings also acknowledged some researchers’ argumentation (e.g., Cheng, 2004; Golombek et al., 2019; Barjesteh and Niknezhad, 2020; Mitchell, 2020). They recognized writing an awkward task due to different writing-specific psychological constructs learners may encounter in the writing process.

This study, in contrast to previous studies, Al-Mekhlafi (2011) and Hashemnejad et al. (2014) found a predictive effect of WSE. In contrast to the findings of this study, they found that there is no interplay between LSE and writing performance. One possible fact may be due to different factors such as cognitive, metacognitive, socio-cognitive, affective factors, and individual differences that affect language learning. As suggested in L2 literature, some researchers (Cheng, 2004; Rankin-Brown, 2006) disentangled some facets of writing anxiety such as somatic anxiety, cognitive anxiety, avoidance behavior, fear of teacher, and class assessment, to name but a few. Other practitioners (Latif, 2007; Tsao et al., 2017; Sabti et al., 2019; Winberg et al., 2019; Barjesteh and Niknezhad, 2020; Cheng, 2020) also have referred to other factors (e.g., linguistic competence, L2 WSE, perceived L2 writing performance, and context of writing) that may provoke L2WA experiences among EFL learners. Examining such dimensions may release different impacts on L2 writing performance.

## Conclusion and Implications

As an attempt to shed more light on the role of learners’ beliefs and self-efficacy in the EFL setting, this study examines the role of two variables (EBs and WSE) in predicting learners’ L2WA in an EFL classroom. Notably, the interplay among the sub-factors of three constructs comprising eight dimensions was probed using SEM modeling. Positive interconnections were observed between two sub-factors of EBs and three sub-factors of L2WA with a mediating role of WSE. Particularly, there is a significant negative relationship between WSE and L2WA, and there is a significant positive correlation between total EBs and L2WA. Overall, it was found that both EBs and WSE had a unique effect on L2WA. However, it was revealed that WSE turned out to be a stronger predictor of L2WA. This implies that the high level of WSE promotes EFL learners’ writing performance. However, the high level of EBs debilitates writing performance. A straightforward conclusion for this study is incorporating a focus on WSE into EFL classrooms, in general, and L2 writing classrooms, in particular. Concerning the pedagogical implications of the current research, L2 teachers should uphold WSE in abating Students’ writing anxiety. Accordingly, material developers and language teachers are suggested to foster learners’ WSE by adopting a practical course of action in writing classrooms with the hope to cope with stressful situations and facilitate writing-specific psychological factors in the writing process.

As the main concerns of this study were based on testing a model, the SEM approach was employed. Therefore, a non-experimental correlational design was adopted due to the nature of the study. As far as the limitations of the study are concerned, it is acknowledged that the findings of such an explanatory study cannot prove causal dependencies. Therefore, in order to obtain more reliable findings on the same constructs, future research design may be employed with experimental groups and control groups to uncover the cause and effect factors. In addition, the generalizability of these findings can be enhanced if future researchers utilize qualitative or mixed-method research designs with different validated scales. Such studies are likely to provide a more in-depth understanding of the predictors of L2WA in the EFL context. Furthermore, this study was delimited only two out of five dimensions suggested by Schommer due to problems with the measurement instrument. Thus, if more reliable and valid measurement instruments exit, the model could be extended.

## Data Availability Statement

The raw data supporting the conclusions of this article will be made available by the authors, without undue reservation.

## Ethics Statement

Ethical review and approval was not required for the study on human participants in accordance with the local legislation and institutional requirements. Written informed consent for participation was not required for this study in accordance with the national legislation and the institutional requirements.

## Author Contributions

All authors listed have made a substantial, direct, and intellectual contribution to the work, and approved it for publication.

## Conflict of Interest

The authors declare that the research was conducted in the absence of any commercial or financial relationships that could be construed as a potential conflict of interest.

## Publisher’s Note

All claims expressed in this article are solely those of the authors and do not necessarily represent those of their affiliated organizations, or those of the publisher, the editors and the reviewers. Any product that may be evaluated in this article, or claim that may be made by its manufacturer, is not guaranteed or endorsed by the publisher.
