# Supplementary material for: Epistemological Beliefs and Writing Self-Efficacy as Predictors of Second Language Writing Anxiety: A Structural Equation Modeling Approach
Source: Front Psychol. 2022 Apr 25;13:850243. doi: 10.3389/fpsyg.2022.850243 (PMC9084311; doi:10.3389/fpsyg.2022.850243)
Supplement: Supplementary file 1 [file Table_1.DOC]

Appendix

The Final items used for the analysis in each Scale

| Scale | Subscales | Items | |
| --- | --- | --- | --- |
| L2WA | Somatic anxiety | 2,6,8,  11,13,  15,19 | 2. I feel my heart pounding when I write English compositions under time constraint.  6. My mind often goes blank when I start to work on an English composition.  8. I tremble or perspire when I write English compositions under time pressure.  11. My thoughts become jumbled when I write English compositions under time constraint.  13. I often feel panic when I write English compositions under time constraint.  15. I freeze up when unexpectedly asked to write English compositions.  19. I usually feel my whole body rigid and tense when I write English compositions. |
|  | Cognitive anxiety | 1,3,7,9,14,17,  20, 21 | 1. While writing in English, I’m not nervous at all.  3. While writing English compositions, I feel worried and uneasy if I know they will be evaluated.  7. I don’t worry that my English compositions are a lot worse than others’.  9. If my English composition is to be evaluated, I would worry about getting a very poor grade.  14. I’m afraid that other students would deride my English composition if they read it.  17. I don’t worry at all about what other people would think of my English compositions.  20. I’m afraid of my English composition being chosen as a sample to be discussed in class.  21. I’m not afraid at all that my English compositions would be rated as very poor. |
|  | Avoidance behavior | 4,5,10,12,16,  18,22 | 4. I often choose to write down my thoughts in English.  5. I usually do my best to avoid writing English compositions.  10. I do my best to avoid situations in which I have to write in English.  12. Unless I have no choice, I would not use English to writ composition.  16. I would do my best to excuse myself if asked to write English compositions.  18. I usually seek every possible chance to write English compositions outside of class.  22. Whenever possible, I would use English to write compositions. |
| WSE | Ideation | 1,2,3,  4,5 | 1. I can think of a lot of original ideas.  2. I can think of many words to describe my ideas.  3. I can think of many ideas for my writing.  4. I know exactly where to place my ideas in my writing.  5.  I can spell my words correctly. |
|  | Conventions | 6,7,8,  9,10 | 6. I can write complete sentences  7. I can punctuate my sentences correctly.  8. I can write grammatically correct sentences.  9. I can begin my paragraphs in the right spots.  10. I can focus on my writing for at least one hour. |
|  | Self-regulation | 11,12,13,14,  15, 16, | 11. I can avoid distractions while I write.  12. I can start writing assignments quickly  13. I can control my frustration when I write.  14. I can think of my writing goals before I write.  15. I know when and where to use writing strategies.  16. I can do what it takes to be a good writer |
| EBQ | Simple/  definitive knowledge | 2,3,4,5,6,7,9,13,14 | 2. Scientists can ultimately get to the truth.  3. Students should seek certain facts and knowledge.  4. A good teacher avoids ambiguity in the course materials.  5. If scientists try hard enough, they can understand the truth to almost everything  6. The best thing about science courses is that most problems have only one right answer.  7. I do not enjoy watching films with confused ending.  9. Working hard on a difficult problem for an extended period of time only pays off for really smart students.  13. Wisdom is not knowing the answers, but knowing how to find the answers.  14. If a person tries too hard to understand problem, they will most likely end up being confused. |
|  | Fast/fixed learning agent | 1,8,  10,1112,1516 | 1. Learning word for word in the definitions is the key to success in exams.  8. Being a good student generally involves memorizing facts.  10. If a person can't understand something within a short amount of time, they should keep on trying.  11. Getting ahead takes a lot of work.  12. People will learn better if they focus more on the process of understanding rather than the facts to be acquired.  15. If you associate the new concepts to your previous knowledge, you will be confused.  16. If one tries hard enough, then one will understand the course material. |
